# The CEACAM1 expression is decreased in the liver of severely obese patients with or without diabetes

**DOI:** 10.1186/1746-1596-6-40

**Published:** 2011-05-11

**Authors:** Wonae Lee

**Affiliations:** 1 Department of Pathology, Dankook University College of Medicine, Cheonan, Korea

## Abstract

**Background:**

Type 2 diabetes is mainly caused by insulin resistance. The carcinoembryonic antigen-related cell adhesion molecule 1 (CEACAM1) is an important candidate for causing insulin resistance.

**Methods:**

The CEACAM1 expression was evaluated immunohistochemically in the liver tissues of 99 severely obese or non-obese subjects with or without diabetes. The CEACAM1 expression was classified into two categories: a normal expression or a decreased expression.

**Results:**

The CEACAM1 expression was markedly decreased in the hepatocytes with macrovesicular steatosis. A decreased CEACAM1 expression was noted in 29 (29%) of 99 cases. The incidence of a decreased CEACAM1 expression was significantly higher in high grade fatty liver as well as severe obesity with or without diabetes (p < 0.05). The incidence of a decreased CEACAM1 expression was not different between the diabetic and non-diabetic groups.

**Conclusions:**

This data supports that a decreased CEACAM1 expression is related to obesity and a fatty liver.

## Background

Diabetes is one of the most problematic diseases in the world, and it is a group of metabolic disease sharing hyperglycemia. Approximately 90-95% of diabetic patients have type 2 diabetes in which insulin resistance predates the development of hyperglycemia and then β-cell dysfunction ensues [1,2]. The molecular cause of insulin resistance is very complicated and this has not yet been clarified. The recent data from articles suggests that carcinoembryonic antigen-related cell adhesion molecule 1 (CEACAM1) is an important candidate molecules that may cause insulin resistance.

CEACAM1 protein is also known as CD66a protein, and the CEACAM1 gene is a human gene that encodes a member of the carcinoembryonic antigen gene family [3,4]. CEACAM1 protein has been implicated in intracellular adhesion, intracellular signaling that governs the growth and differentiation of normal and cancerous cells, and the modulation of immune responses [3,5-8]. CEACAM1 protein is a cell-cell adhesion molecule detected in the pericannalicular region of hepatocytes [9]. CEACAM1 is a liver-specific transmembrane gycoprotein and it is also a substrate of insulin receptor in liver [10,11]. CEACAM1 is phosphorylated in response to insulin in hepatocytes and it plays a role in insulin clearance by increasing receptor-mediated endocytosis [12,13]. Inactivation of CEACAM1 impairs insulin clearance and causes hyperinsulinemia, insulin resistance, dyslipidemia and visceral adiposity in transgenic mice [14]. Thus, there is a strong association between severe visceral obesity, insulin resistance and a reduced hepatic CEACAM1 level [14,15].

The studies that have focused on CEACAM1 related to insulin resistance have mainly been done on experimental animal models and using molecular biologic methods [14,15]. Immunohistochemical studies for determining the CEACAM1 expression have rarely been done in human non-neoplastic liver tissue. The aim of this study is to evaluate the expression of CEACAM1 in the human liver tissue of diabetics and non-diabetics who are with or without severe obesity.

## Methods

### Subjects and samples

A total of 99 cases of liver tissue obtained from diabetic or non-diabetic subjects with or without severe obesity were selected for this study. The age of subjects ranged from 32 to 81 years old (mean: 58.0). Forty cases were obtained from severely obese patients whose body mass index (BMI) ranged from 34 to 67 (mean: 49). They underwent bariatric surgery at the Wakefield hospital in New Zealand. Liver wedge biopsies were done during the gastric bypass surgeries. The average biopsy size was about 2 mm at the greatest dimension. Twenty of 40 severely obese patients had diabetes and 20 of them were non-diabetics. Eight of the 20 severely obese non-diabetics had an abnormal oral glucose tolerance test and they had not yet developed diabetes. Fifty-nine cases were not severely obese patients who were selected from the files of Dankook University Hospital. These patients underwent hepatectomy due to primary or metastatic liver tumor or hepatolithiasis. Among the 59 non-obese patients, 29 patients had diabetes and 30 patients were non-diabetics. Using the paraffin blocks of the hepatectomy specimens, a tissue microarray with 2 mm-sized cores was made. Histologically, all cases were examined for the degree of fatty liver which was graded as low grade = fatty change in more than 5% and less than 33% of hepatocytes and high grade = fatty change in more than 33% of hepatocytes. The severely obese cases were examined for nonalcoholic steatohepatitis (NASH) according to standarized criteria recommended [16]. The liver of non-obese group was not examined for NASH because this group was heterogeneous and inadequate to be assessed for NASH.

### Immunohistochemistry

Immunohistochemical staining was done using the formalin-fixed paraffin-embedded sections. The sections were deparaffinized with xylene and rehydrated with ethanol. Microwave antigen retrieval was performed with 10 mM sodium citrate buffer. The sections were incubated for 20 minutes in 0.3% H_2_O_2 _to block the endogenous peroxidase activity. The sections were then incubated with primary antibody for 60 minutes at room temperature. The primary antibody used was anti-CEACAM-1 mouse monoclonal antibody (1:50, 29H2, Abcam, Cambridge, UK). After washing, the sections were incubated with ImmPRESS™ universal reagent (Abcam) for 40 minutes. Next the sections were incubated with 3, 3'-diaminobenzidine substrate (Zymed, South San Francisco, CA) for 5 min, washed, counterstained with Mayer's hematoxylin, rinsed and mounted.

The CEACAM1 expression was classified into two categories according to the previous report: a normal expression when positive membranous staining was noted throughout the examined liver specimen or a decreased expression when distinct areas of negative staining were noted within the examined liver specimen [16].

### Statistical analysis

Statistical analysis was performed by using the chi-square test. The results were considered to be statistically significant when the p values were less than 0.05. All statistical analyses were conducted using the SPSS 12.0 statistical software program (SPSS, Chicago, IL, USA).

## Results

The relationship of fatty liver and high grade fatty liver with diabetics and severe obesity was summarized in Table 1. Some degree of fatty liver was noted in 63 (64%) of the total of 99 subjects: in 37 (93%) of 40 severely obese subjects and in 26 (44%) of 59 non-obese subjects. The incidence of fatty liver was significantly higher in the severely obese group than the non-obese group (p < 0.05). High grade fatty liver was noted in 35 (35%) of the 99 subjects: in 34 (85%) of 40 severely obese subjects and in 1(2%) of 59 non-obese subjects. The incidence of high grade fatty liver was also significantly higher in the severely obese group than the non-obese group (p < 0.05). The incidence of fatty liver and high grade fatty liver was not different between the diabetes and non-diabetes groups. Twenty four cases (57.5%) of 40 severely obese patients were considered as NASH. All NASH cases were associated with high grade fatty liver.

**Table 1:** Relationship of fatty liver and high grade fatty liver with diabetes and severe obesity

	Total (n = 99)	Fatty liver (n = 63)		High grade fatty liver (n = 35)	
			
		No (%)	p-value	No (%)	p-value
Diabetics					
No	50	30 (60)	0.447	16 (32)	0.481
Yes	49	33 (67)		19 (39)	
Severe obesity					
No	59	26 (44)	<0.0009	1 (2)	<0.0009
Yes	40	37 (93)		34 (85)	

**Figure 1:**
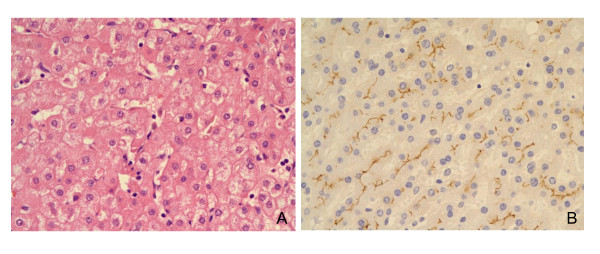
**The liver tissue is not associated with fatty change (A) and it shows positive staining for CEACAM1 alongside the cannalicular membrane of the hepatocytes on immunohistochemistry (B)**.

**Figure 2:**
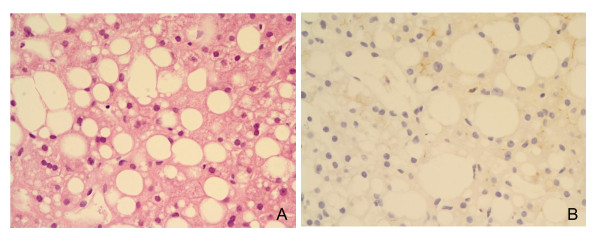
**The liver tissue is associated with severe fatty change (A) and it shows a distinct loss of CEACAM1 expression immunohistochemically (B)**.

CEACAM1 protein was strongly expressed alongside the cannalicular membrane of hepatocytes (Figure 1). The CEACAM1 expression was lost mainly in the hepatocytes with fatty change, particularly macrovesicular steatosis (Figure 2). The relationship of a CEACAM1 expression with diabetes, severe obesity, high grade fatty liver and NASH was summarized in Table 2. A decreased CEACAM1 expression was noted in 29 (29%) of 99 cases. A decreased CEACAM-1 expression was associated with high grade fatty change compared to absent or low grade fatty change (p < 0.05). A decreased CEACAM1 expression was also associated with severe obesity with or without diabetes rather than being associated with non-obesity with or without diabetes (p < 0.05). The incidence of a decreased CEACAM1 expression was significantly higher in severely obese patients with NASH than severely obese patients without NASH (p < 0.05). The incidence of CEACAM1 expression was not different between the diabetic and non-diabetic groups. A decreased CEACAM1 expression was noted in following descending order, severely obese diabetics, severely obese non-diabetics, non-obese diabetics and non-obese non-diabetics (p < 0.05) (Table 3). The relationship of high grade fatty liver, NASH and CEACAM1 expression with severely obese patients that were grouped into diabetics, non-diabetics with abnormal glucose test and non-diabetics with normal glucose test, was summarized in Table 4. There were no statistically significant differences in high grade fatty liver, NASH and a decreased CEACAM1 expression between 3 groups of severely obese patients.

**Table 2:** Relationship of a decreased CEACAM1 expression with diabetes, severe obesity, high grade fatty liver and non-alcoholic steatohepatitis

		Total (n = 99)	Decreased CEACAM1 expression (n = 29) (%)		p-value
Diabetics					
	No	50	13 (26)		0.467
	Yes	49	16 (33)		
Severe obesity					
	No	59	1 (2)		<0.0009
	Yes	40	28 (70)		
High grade fatty liver					
	No	64	0 (0)		<0.0009
	Yes	35	29 (83)		
Non-alcoholic steatohepatitis*					<0.0009
	No	17	6 (35)		
	Yes	23	22 (96)		

* Only 40 severely obese patients was examined for non-alcoholic steatohepatitis

**Table 3:** Relationship of high grade fatty liver and a decreased CEACAM1 expression with diabetics and non-diabetics with or without severe obesity

	Total (n = 99)	High grade fatty liver (n = 35)		Decreased CEACAM1 expression (n = 29)	
			
		No (%)	p-value	No (%)	p-value
Severely obese diabetics	20	18 (90)	<0.0009	15 (75)	<0.0009
Severely obese non-diabetics	20	16 (80)		13 (65)	
Non-obese diabetics	29	1 (3)		1 (3)	
Non-obese non-diabetics	30	0 (0)		0 (0)	

**Table 4:** High grade fatty liver, non-alcoholic steatohepatitis and a decreased CEACAM1 expression between 3 groups of severely obese patients

	Total (n = 40)	High grade fatty liver (n = 34)		Non-alcoholic steatohepatitis (n = 23)		Decreased CEACAM1 expression (n = 28)	
		
		No (%)	p-value	No (%)	p-value	No (%)	p-value
Diabetics	20	18 (90)	0.083	13 (65)	0.412	15 (75)	0.574
Non-diabetics	8	8 (100)		5 (65)		6 (75)	
with abnormal GTT							
Non-diabetics with normal GTT	12	8 (67)		5 (41)		7 (58)	

GTT: glucose tolerance test

## Discussion

Type 2 diabetes is caused by a combination of impaired insulin secretion and insulin resistance, but their relative contribution to the development of hyperglycemia may differ due to the heterogeneity of this disease [2]. In response to physiologic stimuli, insulin is secreted from the pancreatic β-cells into the portal circulation in a pulsatile manner [17]. Through its first passage, approximately 50% of the secreted insulin is cleared in the liver [18,19]. Insulin clearance in liver is a critical regulator of insulin's action [19,20]. Impaired insulin clearance can be the primary cause of insulin resistance by causing downregulation of insulin receptors and hepatic lipogenesis [14,19]. CEACAM1 plays an important role in insulin clearance by receptor-mediated insulin endocytosis and degradation in the liver [12,18]. Abnormalities of insulin clearance are present in various pathological conditions including type 2 diabetes and severe obesity [19].

Obesity is intimately associated with the development of insulin resistance and type 2 diabetes [21-23]. In obese individuals, the adipose tissue releases increased amounts of non-esterified fatty acids, glycerol, hormones, pro-inflammatory cytokines and other factors that are involved in the development of insulin resistance [24-28]. When insulin resistance is accompanied by dysfunction of pancreatic islet beta-cells, this results in the development of diabetes [1,25]. The homeostasis model assessment for insulin resistance (HOMA-R) is strongly related to the waist circumference and BMI [29]. In the present study, 40% of the severely obese non-diabetic group had abnormal oral glucose tolerance testing, and this reflect the presence of insulin resistance, and the remainder of the severely obese group might also be associated with some degree of insulin resistance because they had extremely high BMI. So, the higher incidence of a decreased CEACAM1 expression in severely obese non-diabetic group than non-obese non-diabetic group reflects the possible association between a decreased hepatic CEACAM expression and insulin resistance.

In the present study, the CEACAM1 expression was not different between the diabetics and non-diabetics in the obese or non-obese groups. This result does not support the previous experimental animal model studies [11,12]. It could have been caused by the limitation of immunohistochemical study for conducting a quantitative analysis. In obese group, the CEACAM1 expression was not different between the diabetics and non-diabetics with normal or abnormal glucose tolerance test, yet both the obese diabetic and obese non-diabetic groups had significantly decreased CEACAM1 expression rather than non-obese group. This data suggest the following hypothesis: because a decreased CEACAM1 expression in the obese group is an early event that occurs at the time of insulin resistance and it consistently persists with overt diabetes on a same level, the difference of the CEACAM1 expression between the obese diabetic and obese non-diabetic groups cannot be observed. To clarify this, further study using more a precise analytic method such as Western or Northern blotting is necessary.

Najjar *et al *[12,30] reported that transgenic mice, phosphorylation-defective S503A-CEACAM1 mutant developed visceral obesity with an increased amount of plasma free fatty acid and plasma and hepatic triglyceride content. Subsequently, they reported that CEACAM1 null mice showed the loss of insulin's ability to acutely decrease the hepatic fatty acid synthase (FAS) activity [30]. Generally, insulin is viewed as a positive regulator of fatty acid synthesis by increasing the transcription of FAS mRNA [30]. However, insulin acutely reduces the hepatic FAS activity by inducing phosphorylation of CEACAM1 and its interaction with FAS [30]. This mechanism acts to reduce the hepatic lipogenesis incurred by insulin pulses during refeeding [30]. In the present study, the CEACAM1 expression in the liver was significantly decreased in the severely obese subjects, high grade fatty livers and NASH. Interestingly, the hepatocytes with fatty change, and particularly those with macrovesicular steatosis, revealed the loss of a CEACAM1 expression. This data is in agreement with Najjar *et al *[11,12,14]'s data from experimental animal models.

Liver steatosis is frequently found in type 2 diabetics and in severely obese patients [21,23,31]. One study reported that steatosis was found in 86% of the severely obese subjects who undergo bariatric surgery [32]. In the severely obese group of the present study, 93% of these subjects had an associated fatty liver. Moreover, the degree of fatty liver was mainly high grade. The incidence and grade of fatty liver were not different between the diabetic and non-diabetic groups among the obese groups. This data suggests the hypothesis that a fatty liver is more likely related to the degree of obesity rather than to the blood glucose level in the pathogenesis of fatty liver associated with obese people with or without diabetes.

In conclusion, the CEACAM1 expression was decreased in the liver of severely obese patients with or without diabetes. This study supports that a decreased CEACAM1 expression in liver is related to obesity and a fatty liver. However, the relation between decreased CEACAM1 expression and diabetes was not supported in this study. Larger series with more complex studies are needed to further clarify this.

## Competing interests

The author declares that they have no competing interests.

## Authors' contributions

WAL designed the study, performed literature review, examined immunohistochemistry and prepared the manuscript.
